# Magnitude and associated factors of Atopic dermatitis among children in Ayder referral hospital, Mekelle, Ethiopia

**DOI:** 10.1186/s12895-015-0034-x

**Published:** 2015-08-25

**Authors:** Abraham Getachew Kelbore, Workalemahu Alemu, Ashenafi Shumye, Sefonias Getachew

**Affiliations:** Mekelle University, Tropical Dermatology, Mekelle, North Ethiopia; Dermatovenereology Department, Mekelle University, Mekelle, North Ethiopia; Public Health Department, Mekelle University, Mekelle, North Ethiopia; Addis Ababa University, School of Public Health, Addis Ababa, Ethiopia

**Keywords:** Atopic dermatitis, Cross sectional study, Magnitude, Institutional based study

## Abstract

**Background:**

Atopic Dermatitis (AD) is now a day’s increasing in prevalence globally. A Prevalence of 5–25 % have been reported in different country. Even if its prevalence is known in most countries especially in developing countries there is scarcity with regard to prevalence and associated risk factors of AD among children in Ethiopia settings. The aim of this study was to determine the magnitude and associated factors of atopic dermatitis among children in Ayder referral hospital, Mekelle, Ethiopia.

**Methods:**

A facility-based cross-sectional study design was conducted among 477 children aged from 3 months to 14 years in Ayder referral hospital from July to September, 2014. A systematic random sampling technique was used to identify study subjects. Descriptive analysis was done to characterize the study population. Bivariate and multivariate logistic regression was used to identify factors associated with AD. The OR with 95 % CI was used to show the strength of the association and a *P* value *< 0.05* was used to declare the cut of point in determining the level of significance.

**Results:**

Among the total respondents, 237 (50.4 %) were males and 233 (49.6 %) were females. The magnitude of the atopic dermatitis was found to be 9.6 % (95 % CI: 7.2, 12.5). In multivariate logistic regression model, those who had maternal asthma (AOR: 11.5, 95 % CI:3.3–40.5), maternal hay fever history (AOR: 23.5, 95 % CI: 4.6–118.9) and atopic dermatitis history (AOR: 6.0, 95 % CI:1.0–35.6), Paternal asthma (AOR: 14.4, 95 % CI:4.0–51.7), Paternal hay fever history (AOR: 13.8, 95 % CI: 2.4–78.9) and personal asthma (AOR: 10.5, 95 % CI:1.3–85.6), and hay fever history (AOR: 12.9, 95 % CI:2.7–63.4), age at 3 months to 1 year (OR: 6.8, 95 % CI: 1.1–46.0) and weaning at 4 to 6 months age (AOR: 3.9, 95 % CI:1.2–13.3) were a significant predictors of atopic dermatitis.

**Conclusion:**

In this study the magnitude of atopic dermatitis was high in relation to other studies conducted so far in the country. Maternal, paternal, personal asthma, hay fever histories, maternal atopic dermatitis history, age of child and age of weaning were independent predicators of atopic dermatitis. Hence, the finding alert a needs of strengthening the national skin diseases prevention and control services in particular in skin care of children related to atopic dermatitis and others. In avoiding early initiation of supplementary feeding specially with personal and families with atopic problem needs further attention of prevention activities.

## Background

Atopic dermatitis (AD) is a chronic non-contagious disease that affects the skin. It is characterized primarily by intense itching and the development of papules, scaly lesions, fissures, and crusting [1]. This is one of the most common allergic diseases and manifests as a chronic recurrent dermatitis with itching.

The etiology of AD is multifactorial. Researchers suspect that AD might be caused by environmental factors acting in people who are genetically predisposed to the disease. Heredity is an important biological risk factor in the development of immune sensitization and allergy [2]. Recent data have suggested that loss-of-function genetic variants in the filaggrin gene are associated with AD. Filaggrin plays a role in maintaining the epidermal skin barrier function, whereby it helps to retain moisture in the skin and limits penetration by allergens. These functions can be impaired in filaggrin loss-of-function mutations, this resulting in dry, scaly skin, which increases risk of allergic sensitization and disease [3, 4].

Early life event factors also play a role in the clinical manifestation of AD in children. These may therefore contribute to the increased permeability to foreign proteins in early life and can explain the enhanced antigen uptake in quantities sufficient to influence the immune system this relative intestinal permeability may render the neonate susceptible to pathogen invasion and allergen sensitization [5, 6].

Additionally gastric acid production and enzyme secretion are reduced during the first 4 weeks of life. It is accompanied by immature or disordered intestinal peristaltic activity. The gut-associated lymphoid tissues are incompletely developed at birth. Absorption of large molecules from the gastro intestinal tract (GIT) may lead to immune system dysfunction and, as a result, to the development of AD [5, 7].

The diagnosis of AD is made clinically because there is no laboratory marker or definitive test that can be used to diagnose the condition. Diagnostic criteria for AD were originally developed in an attempt to standardize the type of patient enrolled in research studies. In 1994 a UK Working Party published a minimum list of criteria for AD, which were derived from the Hanifin and Rajka criteria [7, 8].

Studies on the natural history of AD document up to 60 % spontaneous clearing by puberty [9, 10]. AD may recur in adults and the risk is associated with a family history, early onset, severity and persistence of childhood AD and the presence of mucosal atopy.

AD affects up to 20 % of children and 3–5 % of adults in the Western world [11]. The prevalence of AD appear to have risen substantially in many countries in recent decades, a phenomenon that has been attributed variously to changes in lifestyle, nutrition, and other environmental factors. According to the results from a cross-sectional questionnaire survey conducted on random samples of schoolchildren aged 6 to 7 years and 13 to 14 years from centers in 56 countries throughout the world, the prevalence of AD for children aged 6 to 7 years ranged between 2 % (Iran) and 16 % (Japan, Sweden) and for those aged 13 to 14 years ranged between 1 % (Albania) and 17 % (Nigeria). Higher prevalence of AD symptoms was reported in Australia and Northern Europe, and lower prevalence was reported in Eastern and Central Europe and Asia [12].

In Ethiopia in 2005 the prevalence of AD among children age between 1 to 5 years is 4.4 %, which is identified from a cross sectional survey conducted at rural Jimma south west of a country [13].

AD causes various physical problems due to frequent skin damage and itchy sensation, which decrease quality of life. In younger patients, the disease can be sufficiently serious as to disrupt friendships, learning performance, and family relationships, thus negatively influence the overall quality of life in addition to the physical problems. AD has increased in prevalence in many countries in recent decades, but the risk factors for AD in developing countries are unknown [14, 15].

Besides avoiding irritants and moisturizing the skin with emollients, local anti-inflammatory treatment with topical corticosteroids is the mainstay treatment for infants with AD. Parents often fear the side effects and this may lead to non-compliance [16].

The prognosis is reasonable with a recovery rate of 40 % at age two and 65 % in adolescence. AD can be the starting point of the ‘allergic march’, the natural progression of allergic disorders such as asthma and allergic rhinitis. Children with AD have a chance of approximately 40 % to develop asthma [17].

Breastfeeding (BF) and the developing of AD is controversial issue, Human colostrum contains large quantities of secretory IgA (sIgA). Secretory IgA neutralizes infectious agents while at the same time limiting the damaging effects of tissue inflammation that can occur with other antibody types also that can prevent allergen absorption by limiting contact between ingested antigen and the intestinal mucosal membrane [18]. But different studies reports do not support protective effect of BF [19].

Identifying risk factors related to AD is very crucial to prevent the occurrence, recurrence and complications of the diseases among children with strengthening the National Health Service programs on prevention and control of skin diseases. Several risk factors have been identified in the associated with AD among children, of which the three most important are– family history of atopic diseases, early initiation of food, early exposure to antibiotics [1, 2]. In a case of AD various potential socio demographic, environmental, early life event and personal associated risk factors were also investigated in different countries in the world. However, a virtual consensus among AD researchers regarding the fact that family history of atopic diseases, socioeconomic status of family, early use of antibiotics, early exposure to solid foods is a risk factor for AD among children, age, sex, duration of exclusive breast feeding(EBF), have no clear association with AD [2, 13, 14].

Now a day’s the prevalence of AD is increasing in worldwide especially in developing countries of urban area. However, the prevalence and risk factors association with development of AD among children poorly known specifically in Ethiopia [20]. But to the best of our knowledge no study has been done on the magnitude and associated factors of AD among children in Ayder referral hospital, Mekelle in Tigray region. And this study was the first to identify the magnitude and factors associated with AD among children age between 3 months and 14 years who visit dermatology and pediatrics outpatient unit.

## Methods

### Study area and period

This study was conducted in dermatology and Pediatric Outpatient department units of Ayder referral hospital at Mekelle, Ethiopia. Mekelle is the capital city of the Tigray national regional state which is located at 783 km distance north of Addis Ababa. The town had six hospitals (3 private and 3 public), one referral hospital, five health centers, two private higher dermatologic clinics and thirteen higher clinics [21].

Ayder referral and teaching hospital is one of the hospitals which are serving at the Tigray regional state at the north part of Ethiopia since 2007. Presently, the hospital provides various clinical and referral services including dermatological services ranging from primary to specialized care and serves patients referred from different health facilities in Tigray and neighboring regions. It has 500 health professionals working in the hospital with a total of 480 beds for inpatient services [22].

The study was conducted in Ayder referral and teaching hospital from July to September, 2014.

### Study design, study population and sampling

A facility based cross sectional study design was used and children whose age range from 3 months to 14 years who visited the dermatology and pediatrics OPD during the study period were involved.

The sample size was determined by using single population proportion formula with confidence interval 95 % and 3 % margin of error by taking the prevalence rate of AD among children 11.5 % from a study conducted in Mobile Dermatology clinic in Ankober, central Ethiopia [23] and taking the non-respondent rate of 10 % the final sample size becomes 477.

### Sampling procedure

Systematic sampling technique was used to identify the study subjects. In average a minimum of 40 children visit on Monday, Tuesday and Thursday at the Dermatology and Pediatric OPDs of the hospital for treatment seek. On Wednesday and Friday up to 20–25 children visits are expected in the OPDs. In total 165 patients seek the treatment per week (within 5 working days). In this study we included every 2nd child patient coming to the OPDs according to their visit.

### Data collection and quality control

A structure interview questioner was used in the local language once translated from the English version. Additional data was reviewed from clinical examination card of the child patients. The interview was conducted among the mothers or care givers of the children during both OPD visits.

Five trained master of tropical dermatology students were involved in the data collection process and three of them involved as interviewer and the rest two involved in clinical examination and supervision process. The interview was collected using a structured questioner based on the given guide line. The interviewer approached the patient’s mother in a polite and respected manner and kept the confidentiality of patient data.

The socio demographic factors, environmental factors, early life event factors, personal disease associated factors were assessed and skin physical examination was done according to American Academy of dermatology modified paediatrics diagnostic criteria of AD among children. A Pre-test was carried out before actual data collection and some modifications were taken according to the findings. Data completeness and consistency was checked during the collection time and during data entry and cleaning process by doing simple frequency. Ethical clearance was obtained from the Ethical review committee of the College of health science of Mekelle University. Accordingly, permission letter were secured from medical director at Ayder referral hospital. Child Patient identification variables were not used in the study. The studies not inflict harm on or expose children to unnecessary risk as a result of examining of children and interviewing their mothers. Informed consent was obtained verbally from mothers or care givers of children during the interview. When interview and physical examination completed those children who have the problem were linked to the facility for the treatment.

### Data analysis

A descriptive analysis using Proportion and frequency, mean, standard deviation, were used. Bivariate logistic regression was applied to see the association between each independent variable with dependent variable and multiple logistic regression model was used to identify independent predictors. Variable found to be significant at *P value <0.05 *in the bivariate analysis were entered to multiple logistic regression. We used the enter approach in for inclusion into the multivariate model while the Hosmer-Lemeshow statistic was used for model diagnostics. Statistical significance was declared at *P value < 0.05 *and the entered and analysis of the data was performed using SPSS version 20 statistical software package.

### Operational definitions used in the study

**Atopic dermatitis**: patient must have Essential futures with or without important and associated features list according to American Academy of dermatology modified pediatrics AD diagnostic criteria [24].

**Essential features**; are Pruritus and Eczematous changes which must present and, are sufficient for diagnosis: Typical and age-specific patterns and Chronic or relapsing course.

**Important features**: Early age at onset, personal or family atopic history, IgE sensitivity and dryness of skin.

**Associated features**: Keratosis pilaris/Ichthyosis/Palmar hyper linearity, atypical vascular responses, Perifollicular accentuation/Lichenification/Prurigo Ocular/periorbital changes and Perioral/periauricular lesions.

## Result

### Socio-demographic characteristics

In this study a total of 477 children patients, who were enrolled based on the inclusion criteria, are studied. Only 7 children patient’s parents refused to participate, that makes the response rate 98.5 %. Of these participants, 237 (50.4 %) were males and 233 (49.6 %) females, and 341 (72.6 %) were from the urban and 129 (27.4 %) from rural area. The overall mean and standard deviation of age for study participants were 6.63 and (±3.983).

About 133 (28.7 %) fathers of the children attended tertiary school and 111 (23.9 %) of them attended Primary school. whereas, 141 (30.1 %) mothers of the children attend Primary school and 124 (26.4 %) were illiterate. Most of children fathers were 148 (32.0 %) civil servant by occupation. Near to two third 291 (62.2 %) of the children mothers were house wife and 81 (17.3 %) of them were civil servant.

One hundred ninety five (41.5 %) of the family earn a monthly income of less than or equal to 1000 birr and the rest 133 (28.3 %) lie between 1001 and 2000 birr (Table 1).

**Table 1:** Socio-demographic Characteristics of children in Ayder referral hospital, Mekelle, Ethiopia, 2014

Variable	Category	Frequency	Percent
Sex of child	Male Female	237 233	50.4 49.6
Age in category	0.25–1 year 1^+^–5 years 5^+^–10 years 10^+^–14 years	44 168 162 96	9.4 34.5 35.7 20.4
Residence	Urban Rural	341 129	72.6 27.4
Father educational status	Illiterate Read & write Primary school High school Tertiary school completed	50 55 115 111 133	10.8 11.9 24.8 23.9 28.7
Mother educational status	Illiterate Read & write Primary school High school Tertiary school completed	124 28 141 93 83	26.4 6.0 30.1 19.8 17.7
Occupation of Father	Farmer Merchant Civil servant Other	107 111 148 88	23.2 24.0 32.0 20.8
Mother occupation	Civil servant Housewife Merchant Other	81 291 62 34	17.3 62.2 13.2 7.3
Monthly family income	<1000 1001–2000 2001–3000 ≥3001	195 133 65 77	41.5 28.3 13.8 16.4

### Environmental factors

Near to one third of the respondents 169 (36 %) described that the surrounding environment in their living home was open spaces or field and 155 (33 %) mentioned as garden. Among the respondents, 195 (41.5 %) of them had less than or equal to four family sizes and 275 (58.5 %) of them had greater than four. Interview on the number of child siblings shows that 200 (42.6 %) of the respondents had one or no sibling, 222 (47.2 %) had two to three siblings and rest had four or more siblings. Pipe water is the main (84 %) source of drinking water among the respondents of the study. A total of 144 (30.6 %) of the respondents uses insecticide at their home and 56 (11.9 %) of the respondents had exposure to second hand smoking in their living home (Table 2).

**Table 2:** The characteristics of home environmental factors among children in Ayder referral hospital, Mekelle, Ethiopia, 2014

Variable	Category	Frequency	Percent
Home surrounding description	Open spaces or fields nearby Many parks or gardens Few parks or gardens No parks or gardens	169 76 155 70	36.0 16.2 33.0 14.9
People living in the house	<4 >4	195 275	41.5 58.5
Number of siblings	0–1 2–3 4 or more	200 222 48	42.6 47.2 10.2
House type	Single and detached Apartment	390 80	83 17
Roof of house	Corrugated Thatched	437 33	93 7
Floor of house	Mud Cement	211 259	44.9 55.1
Is their carpeted room/s	Yes No	144 326	30.6 69.4
Source of water	Pipe Well River Spring	399 501110	84.9 10.62.32.1
Wood as fuel for cooking	Never Some times Every day	43 133 294	9.1 28.3 62.6
Electricity fuel for cooking	Never Some times Every day	245 137 88	52.1 29.1 18.7
Kerosene fuel for cooking	Never Some times Every day	396 57 17	84.3 12.1 3.6
Animals living in the house	Yes No	301 169	64.0 36.0
Pets contact at 1 year of age	Yes No	245 212	53.6 46.4
Pets contact after 1 year	Yes No	173 282	38.0 62.0
Indoor smoking Exposure	Yes No	56 414	11.9 88.1
Use of insecticide	Yes No	144 326	30.6 69.4

### Early life event factors among children

All of the children had history of breast feeding. Almost near to half 224 (47.7 %) of the children were breast feed for 12 months and above. Three hundred ten (66 %) children have started additional food after age of 6 months and 117 (24.9 %) started at 4 to 6 months age. Animal milk 246 (53.2 %), and packed food and other 160 (34.6 %) were the type of food children exposed before the age of 6 months (Table 3).

**Table 3:** The characteristics of early life event factors among children in Ayder referral hospital Mekelle, Ethiopia, 2014

Variable	Category	Frequency	Percent
Duration of breast feeding	<4 months 4–6 months 7–12 months ≥12 months Do not remember	16 93 125 224 12	3.4 19.6 26.6 47.7 2.6
Exclusive breast feeding duration	<6 months ≥6 months Do not remember	80 370 11	17.3 80.3 2.4
Age first started weaning	<4 months 4–6 months ≥6 months Never Do not remember	20 117 310 12 11	4.3 24.9 66.0 2.6 2.3
Type of food 1^st^ initiated	Animal milk Packed food Other	246 160 56	53.2 34.6 12.1
Vaccination status for any	Yes No	455 15	96.8 3.2
DPT Vaccination	Yes No	455 15	96.8 3.2
Polio Vaccination	Yes No	455 15	96.8 3.2
Child Antibiotic use	Yes No	257 213	54.7 45.3
Fruit intake	Never Once per week More than once per week	71 293 106	15.1 62.3 22.6
Vegetable intake	Never Once per week More than once per week	61 247 162	13 52.5 34.5

### Characteristics of personal and family disease factors

History of asthma is interviewed among the family members and 36 (7.7 %) mothers of the child, 33 (7.0 %) fathers of the child, 11 (2.3 %) siblings and 12 (2.6 %) children themselves had asthma. Among the respondents 52 (11.1 %) of the children had family history of atopy (Table 4).

**Table 4:** Personal and family disease related factors with AD diagnosed according to AAD diagnostic criteria, Mekelle, Ethiopia, 2014

Variable	Category	Frequency	Percent (%)
Family history of atopy	Yes No	52 418	11.1 88.9
Child mother ever had	Asthma Hay fever Atopic dermatitis None	36 12 9 413	7.7 2.6 1.9 87.9
Child father ever had	Asthma Hay fever Atopic dermatitis None	33 17 4 416	7.0 3.6 .9 88.5
Child siblings ever had	Asthma Hay fever Atopic dermatitis None	11 17 6 436	2.3 3.6 1.3 92.8
Child had ever had	Asthma Hay fever None	12 18 440	2.6 3.8 93.6

### Magnitude and distribution of AD with socio demographic characteristics

**Fig. 1:**
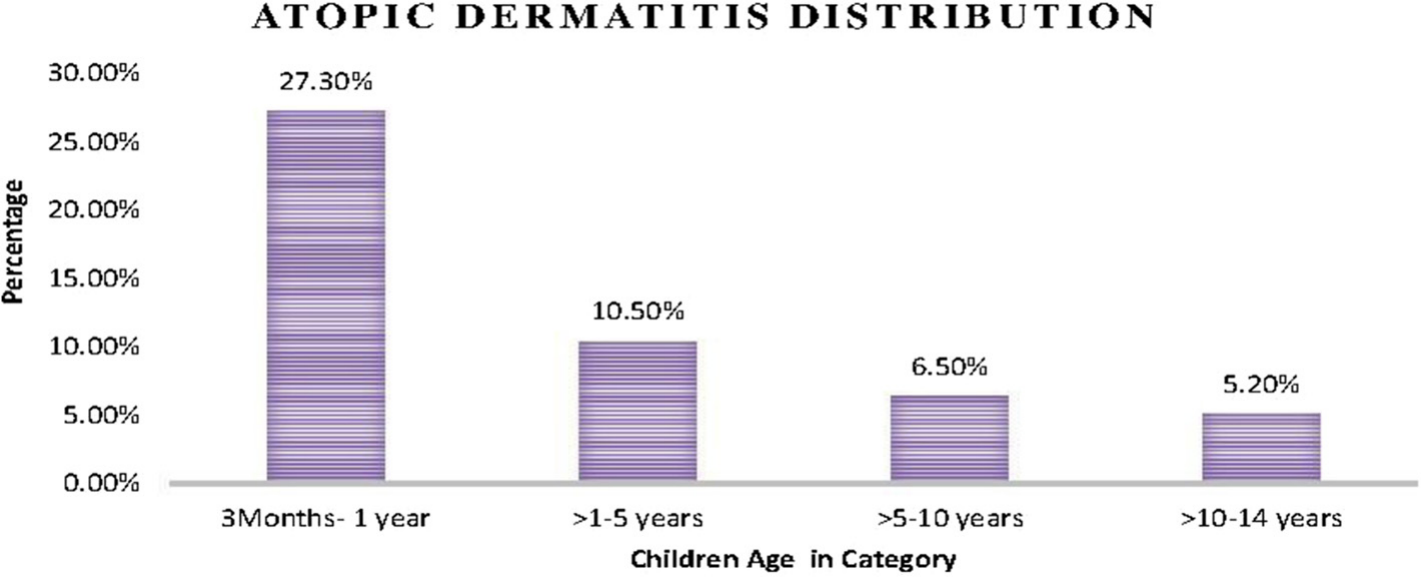
The magnitude of AD according to AAD diagnostic criteria with age category among children in Ayder referral hospital, Mekelle, Ethiopia, 2014

Out of 470 children, 45 of them were diagnosed with AD according to Atopic dermatitis diagnostic (AAD) criteria with a magnitude of 9.6 % (95 % CI: 7.2, 12.5). Among these, 25 (5.3 %) of them were male and the rest 20 (4.3 %) were females. AD was higher among Urban children residence 36 (7.7 %) than rural 9 (1.9 %) children. Among this 20 (4.3 %), 15 (3.2 %), 5 (1.1 %) cases of AD were detected among mothers with house wife status, civil servant, and merchant by occupation. And most of the 19 (4 %) AD diagnosed cases among children were the family monthly income is greater than or equal to 3001birr (~150$). On this study as the age of children increases AD diagnosis were decreased (Fig. 1).

### Distribution of AD among children with home environment and early event factors

**Fig. 2:**
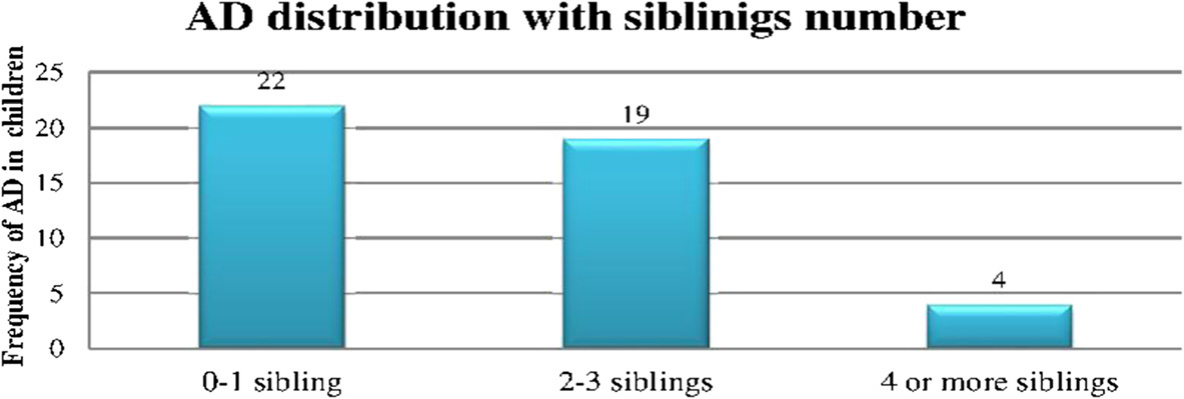
Distribution of AD with number of siblings among children in Ayder referral hospital, Mekelle, Ethiopia, 2014

AD was diagnosed among children whose home surrounding were 20 (4.3 %) open fields followed by few parks and gardens 17 (3.6 %). Children from family size less than four were more diagnosed for AD than those who have greater or equal to four family size. And on segment of children who have one or no siblings 22 (4.7 %) were more diagnosed for AD with followed by two to three siblings 19 (4 %) and above four siblings 4 (0.9 %) (Fig. 2).

### Distribution of AD among children with personal and family disease related characters

**Fig. 3:**
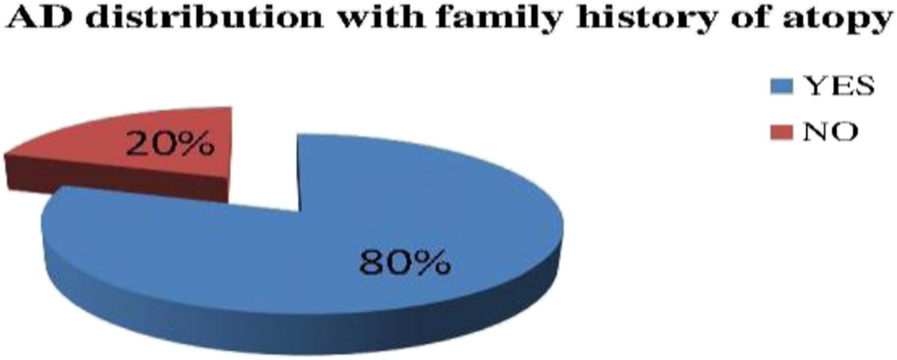
Family history of atopy distribution with AD among children in Ayder referral hospital, Mekelle, Ethiopia, 2014

Children who have AD had maternal history of atopic diseases had asthma, hay fever and dermatitis 37.78, 20 and 11.1 % respectively. The paternal history of atopic diseases shows that asthma (33 %), hay fever (17.78 %), and dermatitis (6 %). Among Children’s who had AD 5 (11.1 %) of them have Personal history of asthma and 6 (13.3 %) had hay fever history. Among AD children 80% of them had family atopy history (Fig. 3).

### Factors associated with atopic dermatitis among children

Comparison of variables that were statistically significant with AD according to AAD diagnostic criteria for AD on crude analysis were adjusted using enter method multivariate logistic analysis model (Table 5). The variables that showed significant association in binary logistic regression model were age, mother education level, father occupation, family monthly income, indoor smoking exposure, use of insecticide at home, exclusive breast feeding, age of weaning, mother atopic diseases, father atopic diseases, siblings atopic diseases status, and personal atopic history.

**Table 5:** Factors associated with atopic dermatitis among children in Ayder referral hospital, Mekelle, Ethiopia, 2014

Variable	Atopic dermatitis		COR (95 % Cl)	AOR (95 % Cl)
	Yes	No		
Age in category				
0.25–1 year	12	32	6.825(2.231–20.883)	6.886 (1.028–46.097)**
1–5 years	17	145	2.134(0.761–5.983)	1.810(0.403–8.123)
5–10 years	11	168	1.275(0.429–3.786)	0.619(0.132–2.917)
10–14 years	5	91	1	1
Mother educational level				
Illiterate	4	46	1	1
Read & write	2	53	1.286(0.252–6.548)	0.165(0.013–2.134)
Primary school	8	107	0.743(0.243–2.273)	0.813(0.168–3.945)
High school	7	104	1.791(0.641–5.000)	2.165(0.397–11.819)
Tertiary school completed	24	109	5.661(2.81–14.052)	2.421(0.363–16.134)
Occupation of Father				
Farmer	6	101	1	1
Merchant	5	106	1.259(0.373–4.256)	1.090 (0.137–8.697)
Civil servant	26	122	0.279(0.11–0.704)	0.866(0.110–6.835)
Other	8	88	0.653(0.218–1.956)	0.841(0.137–5.166)
Monthly family income				
≤ 1000	14	181	1	1
1001–2000	6	127	0.611(0.229–1.632)	0.531(0.093–3.039)
2001–3000	6	59	1.315(0.483–3.576)	0.828(0.145–4.722)
≥ 3001	19	58	4.235(1.999–8.975)	1.799(0.313–10.343)
Indoor smoking Exposure				
Yes	11	45	1	1
No	34	379	0.367(0.174–0.774)	0.845(0.208–3.432)
Use of insecticide				
Yes	27	117	1	1
No	18	308	0.253(0.134–0.477)	0.631(0.210–1.898)
Exclusive breast feed				
< 6 months	13	67	2.199(1.090 4.435)	1.453(0.380 5.558)
> 6 months	30	340	1	1
Age first started weaning				
< 4 months	1	19	0.806(0.102–6.348)	0.571(0.034–9.715)
4–6 months	22	95	3.547(1.840–6.835)	3.965(1.184–13.283)**
> 6 months	19	291	1	1
Child mother ever had				
Asthma	12	24	8.104(3.619–18.15)	11.466(3.246–40.508)**
Hay fever	5	7	11.577(3.420–39.192)	23.492(4.642–118.88)**
Atopic dermatitis	4	5	12.967(3.269–51.433)	5.988(1.007–35.602)**
None	24	389	1	1
Child father ever had				
Asthma	16	17	17.703(7.863–39.859)	13.879(2.439–78.990)**
Hay fever	6	11	10.260(3.459–30.432)	14.432(4.028–51.705)**
Atopic dermatitis	2	2	18.81(2.524–140.172)	5.653(0.794–40.230)
None	21	395	1	1
Child siblings ever had				
Asthma	2	9	2.327(0.48511.164)	7.154 (0.515–99.432)
Hay fever	2	15	1.396(0.308–6.337)	0.685 (0.037–12.566)
Atopic dermatitis	3	3	10.474(2.043–53.700)	2.600(0.223–30.315)
None	38	398	1	1
Child ever had				
Asthma	5	7	8.529(2.570–28.313)	10.495 (1.287–85.552)**
Hay fever	6	12	5.971(2.109–16.902)	12.962 (2.650–63.401)**
None	34	406	1	**1**

NB. ** *P* < 0.05 (significant association)

According to the multiple logistic regression, the odds of AD were 6.9 times higher among children whose age is 3 months to 1 year old than those with age 10–14 years old. (AOR: 6.9, 95 % CI: 1.0–46.1).

The odds of AD were also higher among children those who had history of mother history asthma, allergic rhinitis or hay fever and atopic dermatitis than those children who had no mother history asthma, allergic rhinitis or hay fever and atopic dermatitis. (AOR: 11.5, 95 % CI: 3.3–40.508), (AOR: 23.5, 95 % CI: 4.6, 118.9) and (AOR: 6, 95 % CI: 1.0–35.6) respectively.

The odds of AD were 14 times higher among children those who had history of paternal asthma (AOR: 13.9, 95 % CI: 2.4–79.0). AD was significantly associated with paternal history of Allergic rhinitis or hay fever (AOR: 14.4, 95 % CI: 4.0–51.7).

Personal history of asthma (AOR: 10.5, 95 % CI: 1.3–85.5), allergic rhinitis or hay fever (AOR: 13.0, 95 % CI: 2.7–63.4), weaning (4–6 month) (AOR: 4.0, 95 % CI: 1.2–13.3) were the variables significantly associated with AD among the children.

## Discussion

Atopic dermatitis is one of the most inflammatory skin disease observed among children now a day with increasing prevalence in the world 5–20 % [25]. However, studies on the prevalence and associated risk factor of AD among children are scarce in our country except a few studies conducted. Determining country specific magnitude and Identifying factors related to AD is crucial to halt the occurrence, recurrence and complication of AD by strengthening the national skin diseases prevention and control program. In case of AD among children, various prevalence, demographic, environmental and family and personal diseases factors have been investigated in different countries in the world so far; however, the magnitude and risk factors identified vary based on the (place) countries of studies conducted.

This study identified a magnitude of 9.6 % AD based on the AAD diagnostic criteria. The finding is slightly higher than studies conducted in the country at Ankober mobile clinic which was 4.67 % [23], and at Jimma 4.4 % [13]. However, it is less compared to studies from America 11 % [26], Argentina 41.1 % [27] and Japan 18.6 % [28] but still higher from the Tunisian study of 0.65 % [29]. The difference in the proportion of AD reported among the studies might be due to the geographical difference, use of different diagnostic criteria for diagnosis and the age group considered, study season and period. The possible reason for the higher magnitude compared to studies conducted in Ethiopia could be due to the study setting since the study is done at Ayder hospital which is the only referral hospital providing dermatologic services to patients at Mekelle town in the north part of Ethiopia.

The study identified higher chance of AD among children who had maternal history of asthma, hay fever and atopic dermatitis compared to those children who had no maternal history of asthma, hay fever and atopic dermatitis.

A children who had paternal history of asthma and hay fever was more likely exposed to AD compared to those children who had no paternal history of asthma and hay fever. Personal histories of asthma and hay fever or allergic rhinitis were also the most predicting risk factor for AD.

Maternal, Paternal and personal atopic diseases history is the significantly associated factor with AD in this study and it is consistent with studies done in New Zealand [2], Taiwan [30], Iran [31, 32], Yerevan [33], and South Africa Cape Town [34]. However, it is inconsistent with studies done at rural part of Ethiopia in Jimma area and study from Tunisia [13, 21] which showed no significant association. This difference might be due to methodological differences between the studies, such as different in study design, control of confounding effect and might have information bias which introduced during data collection.

This study was consistent with what has been identified about familial association of AD with atopic family or siblings. So AD is strongly associated with family atopic history factors as documented previously [2].

In children’s age 3 months to 1 year old the odds of AD were almost 7 times higher among children than whose age was 10–14 years old children and it is similar with study done in Korea [35] infants aged up to 1 year 26.5 % were diagnosed with AD. The study revealed that the rate of diagnosis dramatically reduced with increasing age: 11.6, 9.2, and 4.6 % in those aged 3, 5, and 10 years, respectively.

The reduction of AD while age increase after the first year may be the magnitude of AD is greatly affected by changes in the environment in which the overall physical immaturity is expected to be an important factor to influence. Speculated that, immaturity of skin barrier function, mucosal immunity, systemic immunity and digestive enzymes are considered to be factors that influence the development of AD symptoms in infancy [4, 5].

According to this study the odds of AD were about four times higher among children who had early weaning at age 4 to 6 months when compared to those children who had weaning after six months. This is in agreement with study conducted in Sweden [36], Yerevan [33], and Iran [31] and systemic review in European countries [34] which showed a significant association to AD. However, it is in contrary to the findings from Tunisia [29] and Ethiopia at Jimma [13].

This difference can be justified with the use of different age classification across the studies, the study setting and the sample size considered and operationalization of certain variables used in study. The association between early weaning and AD can be explained by the Permeability of foreign proteins of GIT barrier increased during early life period due to the immaturity of digestive system and may render the neonate susceptible to allergen invasion and sensitization [5, 6].

This study found no effect of several risk factors that have long been linked to AD, including sex, residence, education status and occupation of parents, family monthly income, home surrounding, family size, number of siblings, type of house, presence of carpeted rooms at home, source of water, types of fuel used for cooking at home, use of insecticides at home, exposure to indoor smoking, living animals at home, pets contact at 1 year of age and after 1 year, breast-feeding duration, exclusive breast-feeding, vaccination status, antibiotic use, siblings atopic diseases history. However, in other studies variables such as; residence in urban area [37], parent’s education and occupational status, family monthly income, family size, number of siblings [38, 39], breast feeding duration, exclusive breast feeding [28, 33, 36], use of insecticides, exposure to indoor smoking [40] and siblings atopic history [31, 41] were significantly associated with AD among children.

Thus, as it has been explained above, this study is not resistance to some of the differences observed in the empirical literatures related to factors associated with AD. Within the context of this study, some of the results go against some studies and conform to others. Surprisingly, we found some of the variables which had been significant factors of AD in other studies, not statistically significant factors of AD in Ayder referral hospital Mekelle. Nonetheless, based on the availability of resources, the author believes that, the study can be further developed in order to determine magnitude and associated factors of AD at the national level.

## Conclusion

The study found substantially high magnitude of Atopic dermatitis compared to other studies done in the country so far.

Factors such as age of a child, maternal history of asthma, allergic rhinitis/hay fever, Atopic dermatitis, paternal history of asthma and hay fever, personal asthma and hay fever history, Children who started weaning at age of 4 to 6 months were independent predictors of AD among children.

- Hence, these needs great attention through strengthening institutional as well as house hold interventions, targeted towards awareness, prevention and treatment of AD through national skin diseases prevention and control services. Training of health professional particularly health extension workers helps to create awareness about the disease among the communities. Provision of health education to parents coming to the outpatient visit time specifically for those children who have family history of atopic disease is very recommendable. The facility and community based health education still have to address education on care of skin of children and on avoiding early initiation of supplementary feeding to a child and further research work can be recommended to see clear picture at regional or national level.
